# Supplementary material for: Curated and harmonised transcriptomics datasets of interstitial lung diseases
Source: Data Brief. 2025 Oct 14;63:112139. doi: 10.1016/j.dib.2025.112139 (PMC12581653; doi:10.1016/j.dib.2025.112139)
Supplement: Supplementary file 1 [file mmc1.zip › Supplementary_material/RNA-seq/fastQC reports/GSE138283/TRIMMED_SRR10212999_fastqc.html]

TRIMMED\_SRR10212999.fastq.gz FastQC Report 

FastQC Report

Thu 5 Oct 2023  
TRIMMED\_SRR10212999.fastq.gz

## Summary

- Basic Statistics
- Per base sequence quality
- Per sequence quality scores
- Per base sequence content
- Per sequence GC content
- Per base N content
- Sequence Length Distribution
- Sequence Duplication Levels
- Overrepresented sequences
- Adapter Content

## Basic Statistics

| Measure | Value |
| --- | --- |
| Filename | TRIMMED\_SRR10212999.fastq.gz |
| File type | Conventional base calls |
| Encoding | Sanger / Illumina 1.9 |
| Total Sequences | 91780916 |
| Sequences flagged as poor quality | 0 |
| Sequence length | 60-76 |
| %GC | 50 |

## Per base sequence quality

## Per sequence quality scores

## Per base sequence content

## Per sequence GC content

## Per base N content

## Sequence Length Distribution

## Sequence Duplication Levels

## Overrepresented sequences

| Sequence | Count | Percentage | Possible Source |
| --- | --- | --- | --- |
| CCTTAGGCAACCTGGTGGTCCCCCGCTCCCGGGAGGTCACCATATTGATG | 281361 | 0.30655719321868613 | No Hit |
| CTCCGTTTCCGACCTGGGCCGGTTCACCCCTCCTTAGGCAACCTGGTGGT | 258120 | 0.28123493559380036 | No Hit |
| CCCCTCCTTAGGCAACCTGGTGGTCCCCCGCTCCCGGGAGGTCACCATAT | 209450 | 0.22820648248923556 | No Hit |
| CCAGGCTGGAGTGCAGTGGCTATTCACAGGCGCGATCCCACTACTGATCA | 206914 | 0.22544338084400903 | No Hit |
| CTTCGATGTCGGCTCTTCCTATCATTGTGAAGCAGAATTCACCAAGCGTT | 187590 | 0.2043888949637417 | No Hit |
| CTCAGGCTGGAGTGCAGTGGCTATTCACAGGCGCGATCCCACTACTGATC | 148632 | 0.1619421623554073 | No Hit |
| GGGAGTTTGACTGGGGCGGTACACCTGTCAAACGGTAACGCAGGTGTCCT | 147687 | 0.16091253654517895 | No Hit |
| GTTGGATTGTTCACCCACTAATAGGGAACGTGAGCTGGGTTTAGACCGTC | 143129 | 0.15594636253140032 | No Hit |
| CCCTCCTTAGGCAACCTGGTGGTCCCCCGCTCCCGGGAGGTCACCATATT | 132004 | 0.1438251062998761 | No Hit |
| CAGGCTGGAGTGCAGTGGCTATTCACAGGCGCGATCCCACTACTGATCAG | 118531 | 0.1291455840340491 | No Hit |
| CCTCCTTAGGCAACCTGGTGGTCCCCCGCTCCCGGGAGGTCACCATATTGATGCCGAACTTAGTGCGGACACCCG | 110094 | 0.1199530412182855 | No Hit |
| CTTAGGCAACCTGGTGGTCCCCCGCTCCCGGGAGGTCACCATATTGATGC | 108481 | 0.11819559525860474 | No Hit |
| CTCCTTAGGCAACCTGGTGGTCCCCCGCTCCCGGGAGGTCACCATATTGA | 100258 | 0.10923621638293521 | No Hit |

## Adapter Content

Produced by FastQC (version 0.11.7)
